# Study of Autophagy and Microangiopathy in Sural Nerves of Patients with Chronic Idiopathic Axonal Polyneuropathy

**DOI:** 10.1371/journal.pone.0163427

**Published:** 2016-09-23

**Authors:** Kristin Samuelsson, Ayman A. M. Osman, Maria Angeria, Mårten Risling, Simin Mohseni, Rayomand Press

**Affiliations:** 1 Department of Clinical Neuroscience, Department of Neurology, Karolinska Institutet, Karolinska University Hospital, Stockholm, Sweden; 2 Department of Clinical and Experimental Medicine, Division of Cell Biology, Linköping University, Linköping, Sweden; 3 Department of Neuroscience, Karolinska Institutet, Stockholm, Sweden; Szegedi Tudomanyegyetem, HUNGARY

## Abstract

Twenty-five percent of polyneuropathies are idiopathic. Microangiopathy has been suggested to be a possible pathogenic cause of chronic idiopathic axonal polyneuropathy (CIAP). Dysfunction of the autophagy pathway has been implicated as a marker of neurodegeneration in the central nervous system, but the autophagy process is not explored in the peripheral nervous system. In the current study, we examined the presence of microangiopathy and autophagy-related structures in sural nerve biopsies of 10 patients with CIAP, 11 controls with inflammatory neuropathy and 10 controls without sensory polyneuropathy. We did not find any significant difference in endoneurial microangiopathic markers in patients with CIAP compared to normal controls, though we did find a correlation between basal lamina area thickness and age. Unexpectedly, we found a significantly larger basal lamina area thickness in patients with vasculitic neuropathy. Furthermore, we found a significantly higher density of endoneurial autophagy-related structures, particularly in patients with CIAP but also in patients with inflammatory neuropathy, compared to normal controls. It is unclear if the alteration in the autophagy pathway is a consequence or a cause of the neuropathy. Our results do not support the hypothesis that CIAP is primarily caused by a microangiopathic process in endoneurial blood vessels in peripheral nerves. The significantly higher density of autophagy structures in sural nerves obtained from patients with CIAP and inflammatory neuropathy vs. controls indicates the involvement of this pathway in neuropathy, particularly in CIAP, since the increase in density of autophagy-related structures was more pronounced in patients with CIAP than those with inflammatory neuropathy. To our knowledge this is the first report investigating signs of autophagy process in peripheral nerves in patients with CIAP and inflammatory neuropathy.

## Introduction

Polyneuropathy is a common neurological condition due to disturbed function of the peripheral nerves. The prevalence of polyneuropathy is 1.66% [1], though the incidence increases with age [2], being as high as 6.6% among the population over sixty years of age [1]. Polyneuropathy is a heterogeneous disease with regards to underlying etiology, and 25% of polyneuropathies (commonly termed as chronic idiopathic axonal polyneuropathy, CIAP) are judged to be idiopathic [2, 3].This makes CIAP one of the most prevalent non-treatable neurological disorders known today, with an estimated prevalence of 5–8 million people in the US [4]. Chronic idiopathic axonal polyneuropathy is a slowly progressive predominantly sensory axonal polyneuropathy with an insidious onset, relatively low level of disability, and per definition no identifiable cause [5–9]. Arguments have been made for an underlying vascular /metabolic cause for CIAP, though no single metabolic factor has been consistently identified by different research groups [10–19].

In contrary to the metabolic arguments, somewhat stronger arguments have been made in implicating microangiopathy in the vasa nervorum of peripheral nerves as the pathogenic cause of CIAP. Structural abnormalities in the endoneurial microvessels indicating microangiopathy, such as increased basal membrane area or basal lamina area thickness (BLAT), reduced lumen area, increased endothelial cell area, increased number of endothelial cell nuclei with increased basal lamina area as the most consistent finding, are well documented in sural nerve biopsies from patients with diabetes mellitus [20–24]. Similar changes have been shown in patients with atherosclerotic peripheral vascular disease without diabetes as an indication of attribution of chronic ischemia for the pathogenesis of the structural abnormalities [25]. Patients with CIAP and diabetic neuropathy share several clinical and electrophysiological hallmarks, so it is perceivable that microangiopathy could be the main cause of axonal damage even in CIAP. However, there is very limited data available on histopathological characterization of peripheral nerves in patients with CIAP. Another possible contributing factor to the pathogenesis of CIAP may be age-related axonal degeneration causing disturbed axonal structure or function, as suggested by the fact that the incidence of CIAP correlates positively with age. An alternative cause of axonal degeneration in peripheral nerves in CIAP may be genetic mutations in axonal genes. This notion is supported by the identification of the mutation in the gene coding for neurofilament light chain, causing disruption in the axonal cytoskeleton and leading to hereditary motor sensory neuropathy (HMSN) type 2E [26].

Autophagy is a cellular process responsible for clearance of damaged organelles and macromolecules. This recycling pathway is a normal process in the cell and has a critical role in the cellular homeostasis. Dysfunction in the autophagy pathway has been suggested to contribute to several neurodegenerative conditions in the CNS such as Huntington’s, Parkinson’s, Alzheimer’s diseases and amyotrophic lateral sclerosis [27], as well as in muscle diseases such as Danon disease [28], X-linked myopathy with excessive autophagy [29], drug-induced autophagic vacuolar myopathies [30], sporadic inclusion body myositis [31] and Hereditary Inclusion Body Myositis with Paget’s disease [32]. The autophagy process in the peripheral nervous system is not yet thoroughly explored. Osman et al recently showed that the autophagy pathway exists in human peripheral nerve i.e. in the posterior interosseous nerve, and suggested that there was a difference in manifestation of this pathway in patients with type 1 vs. type 2 diabetes [33]. To our knowledge there are no reports of ultra-structural studies of autophagy-related structures (ARS) in patients with CIAP.

In the present study we intended to explore the presence of microangiopathy in patients with CIAP in comparison to controls without sensory polyneuropathy and patients with inflammatory neuropathy. We also aimed to find out whether the density of ARS in the peripheral nerves of patients with CIAP differs from that observed in the other groups.

## Materials and Methods

### Patients and Controls

Fascicular sural nerve biopsies obtained from 10 patients with idiopathic axonal sensorimotor polyneuropathy, and age and gender-matched controls consisting of 11 patients with inflammatory neuropathy and 10 controls without sensory polyneuropathy were used for this study. The biopsies of patients with idiopathic sensorimotor axonal polyneuropathy and inflammatory neuropathy had been obtained by Dr. R. Press during the period 2002–2009, whereas biopsies from normal controls had been obtained by Dr. Solders during the period 1981–1986. The electrophysiological investigation of all patients with idiopathic axonal neuropathy, chronic inflammatory demyelinating polyneuropathy (CIDP) and vasculitic neuropathy had been performed at the Department of Neurophysiology at Karolinska University Hospital.

All ten patients with idiopathic sensorimotor axonal neuropathy had been examined at our tertiary neuromuscular center and the biopsies were part of the etiologic investigation of patients with unknown cause for an axonal sensorimotor polyneuropathy, where the clinician had deemed it necessary to rule out isolated peripheral nervous system (PNS) vasculitis and/or amyloidosis.

Controls with inflammatory neuropathy consisted of six patients with CIDP and five patients with isolated PNS vasculitic neuropathy. The six patients with CIDP fulfilled the electrophysiological and clinical EFNS 2006 criteria for Probable CIDP, though retrospectively for the patients biopsied during the period 2002–2006 [34]. The reason for obtaining sural nerve biopsies from these six patients with CIDP was the fact that they had previously not completely fulfilled all the diagnostic criteria for CIDP, usually with some electrophysiological CIDP parameter lacking. The five samples with vasculitic neuropathy fulfilled the histopathological criteria for vasculitis, as judged by an experienced neuropathologist [35].

This retrospective study, using material from the Biobank, was approved by the local ethical committee in Stockholm, Dnr. 2011/104-31/4, Dnr. 2013/1754-32/4.

All biopsies had been stored in the Biobank at the Department of Clinical Pathology, Karolinska University Hospital, Huddinge following the biopsy procedure. Written consent was not obtained at the time-point of the biopsy procedure since the sural nerve biopsies in all cases (patients and controls) were performed as part of an ongoing clinical investigation at the Department of Neurology. The oral informed consent to save excess sural nerve tissue in the Karolinska Biobank after all diagnostic procedures had been performed on obtained nerve tissue was collected by the treating physician at that time. The patients were informed orally about the standard procedure at that time, i.e. the fact that the excess tissue material could be used in future analyzes. Written consent was not obtained afterwards for this specific study since this is a strictly retrospective study only using material from the Biobank. No additional sampling or investigations of the patients/controls were performed. According to the current Swedish Act on Biobanks (applicable since 2002) the patients consent to save biopsy material is documented on each pathology referral form. For the biopsies taken pre 2002 no written documentation was performed according to the regulations in Sweden at that time.

### Inclusion Criteria for Sural Nerve Biopsies

#### Inclusion Criteria for CIAP Sural Nerve Biopsies (1-4 fulfilled)

1. Adult patients with electrophysiological signs of an axonal, predominantly distal, sensorimotor neuropathy.
2. No underlying cause of neuropathy identified through an extensive interview with the patient and laboratory work-up of known causes of neuropathy.
3. No histopathological signs of inflammation, including vasculitis, or of amyloidosis detected in the sural nerve biopsy.
4. No clinical evidence of vasculitic neuropathy, amyloidosis or any other underlying etiology seen upon long-term follow-up.

#### Inclusion Criteria for Inflammatory Neuropathy Sural Nerve Biopsies (1-4 fulfilled)

1. Adult patients with a clinical and electrophysiological phenotype of either Probable CIDP according to the 2006 EFNS criteria, or isolated PNS vasculitic neuropathy.
2. No alternative underlying causes of neuropathy identified through a standardized laboratory work-up.
3. Sural nerve biopsy showed histopathological signs either of demyelination according to the EFNS CIDP criteria 2006 (six patients), or of vasculitic neuropathy (five patients).
4. No clinical evidence other than CIDP or of isolated PNS vasculitic neuropathy was seen upon long-term follow-up in the six CIDP and five vasculitic neuropathy patients respectively.

#### Inclusion Criteria for Sural Nerve Biopsies from Controls without Sensory Polyneuropathy

Sural nerve biopsies obtained from 10 individuals, given other diagnoses than sensory polyneuropathy on follow-up examination, were used. At the time when these 10 patient controls were clinically investigated at our tertiary neuromuscular center, sural nerve biopsies were performed in almost all patients with symptoms of distal weakness and/or sensory symptoms from the lower limbs. The sural nerve biopsy was hence a routine procedure in an early disease state when suspecting polyneuropathy, as part of the assessment of the peripheral nerves. The biopsies have been used as normal controls in earlier publications [36–38].

### Experimental Design

This was a retrospective study. The specimens were coded and mixed randomly by the laboratory assistant preparing the semi-thin sections prior to histopathological analysis. The slides were given a random number 1–31. The work at the light and electron microscope as well as the analysis on micrographs was performed on coded specimens. The software program ImageJ (http://imagej.nih.gov/ij/) was used for all measurements and calculations.

### Biopsies

A fascicular sural nerve biopsy was performed in local anesthesia from the area posterior to the lateral malleolus. The numbers of contained fascicles were one to nine. One part of the sample was fixed in 2% glutaraldehyde in Sörensen´s phosphate buffer, pH 7.4, for one day and post-fixed in osmium tetroxide for two hours and embedded in Epon. Ultratome V (LKB, Sweden) was used to prepare transverse semi-thin (0.5 μm) sections for light microscopy. The sections were stained with 1% toluidine blue. Ultratome (Leica Microsystems, Germany) was used to prepare ultra-thin sections (60 nm) for electron microscopy (EM). The sections were then contrasted with 10% uranyl acetate for 20 minutes followed by 0.4% lead citrate for 3 minutes. All nerve biopsies had been previously scrutinized by an experienced neuropathologist in a clinical setting.

### Analysis of Semi-Thin Sections

Digital images were taken at different magnifications by using a Nikon eclipse E600 light microscope equipped with a Nikon Digital Sight U1 camera. The following objectives were used: 20x (fascicle area), 40x (identifying microvessels) and 60x (fiber counting and measurements of myelinated axon diameters).

#### Capillary Density

Direct calculation of endoneurial and subperineurial microvessels was performed from micrographs covering the total fascicular area from each sample (no. of vessels counted 5–45). There was no significant difference in the total number of vessels counted between the three groups. The density was calculated by dividing the numbers of vessels by the fascicle area (numbers/mm^2^).

#### Myelinated Fiber Density and Diameter

Myelinated fibers were counted in three randomly taken micrographs covering different areas and if possible from different fascicles (no. of fibers counted 15–993). The density was calculated by taking the total numbers of fibers from each sample and dividing it by the area covered in the micrographs (numbers/mm^2^). For calculation of fiber diameter, each micrograph was divided in four quadrants. The diameters of the fibers in the upper quadrant in each micrograph were determined by measuring the perimeter of each fiber; calculations were then performed by using ImageJ. For some samples with very few fibers all myelinated fibers were included in the assessment of the diameter (no. of fibers measured 10–97). Only fibers that had a circular or ovoid form with clearly visible axons were included in quantifications.

### Analysis of Ultrathin Sections

For ultra-structural studies, the JEOL JEM-1200EX transmission electron microscope (JEOL, Peabody, MA, USA) was used.

#### Unmyelinated Fiber Density and Diameter

Twenty five micrographs with 15,000x magnification were prepared for each sample. The photography was initiated at one corner of the fascicle, and continued at a rate of one picture from every third microscopic field. The numbers of fibers in each micrograph were counted and diameter of each fiber was measured. Density of unmyelinated axons was calculated by dividing the number of axons with the area of the micrographs (number/mm^2^) (no. fibers counted 9–109).

#### Density of Autophagy-Related Structures

In the 25 micrographs mentioned above direct calculation of ARS was performed. The structures that were counted were dense osmophilic lysosomes, phagophores, autophagosomes, and autolysosome-like structures [33]. One sample from the inflammatory neuropathy group was excluded from the analysis due to technical issues.

#### Parameters of Microangiopathy

All endoneurial and subperineurial vessels identified in the EM sections were photographed at a magnification of 2,500x -12,000x adjusted to the size of the vessel. The vessels were excluded if the ratio between the largest and smallest diameter differed more than 3:1 (indicating a blood vessel cut obliquely), if they were entirely surrounded by a periendothelial cell layer (indication of a larger vessel), or if they in reevaluation were judged to be located in the perineurium. In total 206 vessels were photographed. Thirty three vessels were excluded due to above mentioned reasons as well as technical difficulties. The numbers of included vessels per sample varied from one to nine. There was no significant difference between the number of vessels per sample included in the study between the patients and the two control groups.

Circumferences of the lumen, the endothelial cell layer and the total vessel including basal lamina were drawn on the images and the areas were calculated by the software program. The area of the endothelial cell layer was calculated by subtracting the lumen area from the lumen and endothelial area. The basal lamina area was calculated by subtracting the lumen and endothelial area form the total vessel area. The basal lamina area thickness (BLAT) was calculated by subtraction of the radius of a circle equivalent of lumen and endothelial area (LEA), from the radius of a circle equivalent of the total vessel area (VA), (BLAT = √VA/π-√LEA/π) [39]. The numbers of endothelial- and the periendothelial cell nuclei were calculated in every vessel. We use the term periendothelial cell since it sometimes was difficult to differ between a primitive smooth muscle cell and a pericyte; the term has been used in earlier publications [40, 41].

For assessment of the endothelial cell profile the number of endothelial intercellular junction was counted in each microvessel in micrographs of higher magnification 8,000x-60,000x.

### Review of Medical Records

Medical records for CIAP patients and inflammatory controls were reviewed for identification of risk factor profiles for cardiovascular disease, as well as electrophysiological data.

### Statistical Analysis

All data is presented in medians and interquartile range if not noted otherwise. The Kruskal-Wallis multiple comparison test for non-parametric values was used for comparisons between groups. For direct comparison between two groups Mann-Whitney U, two-tailed test for non-parametric values and unpaired t-test for comparing parametric values were used. The alpha-level set for significance was 0.05. Spearman’s rank correlation was used for correlation analysis. For contingency table the Fisher’s exact test, two-tailed, was used. Statistical analyses were performed using GraphPad Prism 6 (GraphPad Software Inc, San Diego, USA).

## Results

### Demographics and Clinical Data

The demographic data is presented in Table 1. The normal controls were somewhat younger and had a male predominance compared to the index group with CIAP, but the differences were not significant. The duration of symptoms prior to the biopsy was as expected longer for the patients with CIAP compared to the patients with inflammatory neuropathy. There were no significant differences between the groups regarding risk factors for vascular disease such as smoking, metabolic and cardiovascular diseases, or other clinical parameters such as pain and the need for a walking aid.

**Table 1:** Demographic, Clinical and Electrophysiological Data for Patients with CIAP, Inflammatory Neuropathy and Normal Controls.

		CIAP *n = 10*	Inflammatory neuropathy *n = 11*	Normal controls *n = 10*	
**Age**	**mean**	54.9	51.4	45.7	ns
	**median**	57	55	50	ns
	**range**	25–78	39–59	18–61	
**Sex, m:f**		5:5	6:5	7:3	ns
**Smokers, y:n**		4:6	3:7^b^	^a^	ns
**Metabolic and cardiovascular comorbidities, y:n**^**c**^		3:7	4:7	^a^	ns
**Duration of disease (years)**	**mean**	6.5	3.3	^a^	ns
	**median**	5.0	1.5	^a^	*p<0*.*05*
	**range**	2–19	0.1–14	^a^	
**Severity of gait disorder; need for walking aids, y:n**		2:8	3:8	^a^	ns
**Neuropathic pain, y:n**		5:5	6:5	^a^	ns
**Electroneurography index**^**d**^	**mean**	2.52	5.10^e^	^a^	ns
	**median**	2.03	3.35^e^	^a^	ns
	**range**	1.26–6.90	0.38–11.40	^a^	

CIAP = chronic idiopathic axonal polyneuropathy

^a^Data not available

^b^Data lacking for one patient

^c^Metabolic and cardiovascular comorbidities comprised of hypertension, hypercholesterolemia and moderate levels of white matter changes/ischemia lesions on brain CT/MRI.

^d^Electroneurography index is a composite index based on 12 electrophysiological parameters (conduction velocity, F-latency and amplitude) from five sensory and motor nerves from the upper and lower extremities. Normal value for index is 0.72 [42].

^e^Data lacking for two patients, n = 9

Nerve conduction studies showed a predominant sensory or sensorimotor axonal polyneuropathy in all 10 patients with CIAP. The nerve conduction studies indicated that the patients with inflammatory neuropathy were more severely affected than the patients with CIAP, although there was no significant difference in the electrophysiological index between the two groups (Table 1). The sural nerve amplitude was absent in five of 10 patients with CIAP, and abnormally low (1–6 μV) in relation to age and height in four patients with CIAP. Data for sural nerve amplitude was lacking for one patient with CIAP.

The clinical and electrophysiological phenotypes did not match in all CIAP cases. Six of the 10 patients with CIAP had a predominantly sensory clinical phenotype, two patients had sensorimotor symptoms and two patients had predominantly distal motor symptoms. However, electroneurography showed a length-dependent affection of both sensory and motor nerves in all 10 patients with CIAP. All five patients with CIAP who had neuropathic pain had signs of small fiber affection upon quantitative sensory testing.

### Light Microscopy Analysis

**Fig 1:**
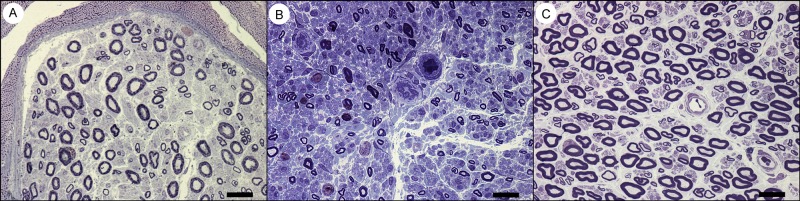
Light Microscopy Pictures from Patients with CIAP, Inflammatory Neuropathy and Normal Controls. Light microscopy pictures from (a) a patient with CIAP, (b) inflammatory neuropathy and (c) a normal control. All scale bars = 20μm.

**Fig 2:**
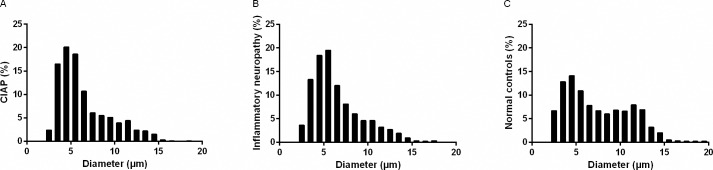
Size Distribution of Myelinated Fibers in Sural Nerves. Size distribution of myelinated fibers in sural nerves of patients with (a) CIAP, (b) inflammatory neuropathy and (c) normal controls.

There was a significant loss of large myelinated fibers both in patients with CIAP (*p*<0.05) and inflammatory neuropathy (p<0.001) compared to the normal controls; fibers with a diameter > 10 μm in CIAP, inflammatory neuropathy and normal controls constituted 15%, 14%, and 28% of the fibers, respectively (Table 2; Figs 1 and 2). Size distribution of myelinated fiber diameter showed a bimodal pattern in the controls with peaks at 4–5 μm and 11–13 μm and a unimodal pattern in CIAP and inflammatory neuropathy groups (peaks at 4–6 μm). There was no significant difference in the capillary density between the three groups.

**Table 2:** Light and Electron Microscopy Data of Sural Nerve Biopsies Obtained from Patients with CIAP, Inflammatory Neuropathy and Normal Controls.

	CIAP *n = 10*	Inflammatory neuropathy *n = 11*	Normal controls *n = 10*
**Myelinated fiber density (no/mm**^**2**^**)**	5,972* (4,186–6,835)	3,113*** (1,506–6,245)	8,648 (7,575–10,337)
**Capillary density (no/mm**^**2**^**)**	83.9 (63.1–107.7)	60.0 (48.0–77.2)	70.4 (56.7–101.4)
**Myelinated fiber diameter (μm)**	5.6**** (4.3–8.2)	5.7**** (4.5–8.0)	6.7 (4.4–10.5)
**Unmyelinated fiber density (no/mm**^**2**^**)**	26,937 (9,943–42,976)	30,364 (19,990–42,346)	35,143 (23,996–42,145)
**Unmyelinated fiber diameter (μm)**	1.15 (0.9–1.4)	1.01**** (0.7–1.3)	1.15 (0.9–1.4)
**BLAT (μm)**	2.7 (1.8–3.5)	3.3** (2.3–4.5)	2.5 (1.9–3.2)
**Basal lamina area (μm**^**2**^**)**	81.0 (49.4–116.3)	114.1** (71.0–203.5)	82.8 (54.1–111.2)
**Lumen area (μm**^**2**^**)**	7.0 (2.3–16.2)	7.5 (1.6–22.7)	11.8 (3.6–27.1)
**Endothelial cell area (μm**^**2**^**)**	28.2 (18.6–35.2)	46.5**** (29.0–78.3)	25.8 (17.4–35.1)
**Endothelial cell nuclei (no)**	1.5 (1.0–1.9)	2.5 (1.3–3.2)	1.5 (1.2–1.7)
**Endothelial cell profile (no)**	5.4 (4.8–6.2)	6.8* (5.6–8.2)	5.2 (4.6–5.8)
**Periendothelial cell nuclei (no)**	0.7 (0.6–0.9)	1.4*** (1.0–1.8)	0.6 (0.3–0.8)
**Autophagy-related structures (no/mm**^**2**^**)**	9,475** (5,500–15,028)	7,114*^a^ (6,021–10,245)	4,244 (2,078–6,283)

CIAP = Chronic idiopathic axonal polyneuropathy

BLAT = Basal lamina area thickness

Values are presented as median and the 25^th^-75^th^ percentiles

*p<0.05

**p<0.01

***p<0.001

****p<0.0001 vs. healthy controls

^a^Data lacking for one inflammatory control

### Qualitative Description from Observations of Sural Nerves in the Electron Microscope

#### General Comments

Glycogen granules in the cytoplasm of different cell types particularly Schwann cells, π-granules and zebra bodies, as well as a few large unmyelinated axons (> 2 μm in diameter) were observed in all samples irrespective of underlying diagnosis.

#### Patients with Chronic Idiopathic Axonal Polyneuropathy

**Fig 3:**
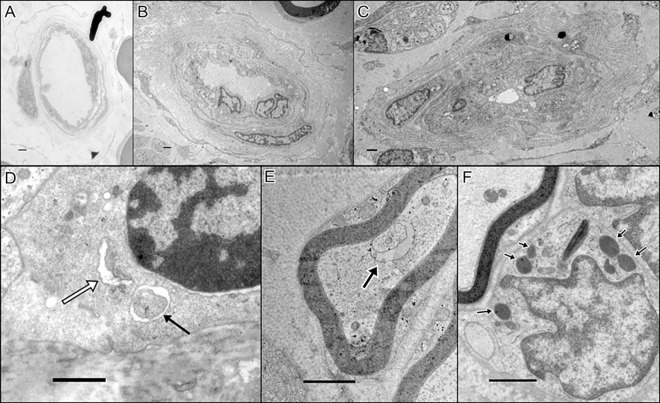
Electron Microscopy Pictures of Endoneurial Vessels and of Autophagy-Related Structures. An endoneurial vessel from (a) a normal control, (b) a patient with chronic idiopathic axonal polyneuropathy with a prominent basal lamina area thickness, and (c) a patient with a vasculitic neuropathy with a reduplication of basal lamina. (d) Phagophore (white arrow) and autophagosome (black arrow) in a Schwann cell in a patient with inflammatory neuropathy. (e) Autophagosome in a myelinated axon (arrow) in a patient with CIAP. (f) Lysosomes in a Schwann cell (arrows) in a patient with inflammatory neuropathy. All scale bars = 1 μm.

The histology/pathology of the nerve samples in this group varied inter-individually from being normal to showing extensive loss of large and medium-sized axons. Ongoing axonal degeneration of myelinated fibers, and the presence of numerous lysosomes and autolysosome-like structures in the unmyelinated axons and their associated Schwann cells (Fig 3), comprised the pathological hallmark in CIAP; Lysosomes were mostly observed in unmyelinated axons and their associated Schwann cells, and phagophore-like structures were mostly common in unmyelinated axons. Only few ARS [phagophores, autophagosomes, lysosomes and autolysosomes] were observed in myelinated axons or their associated Schwann cells. Collagen pockets and bands of Büngner were observed in all samples, but well-developed regeneration units including regenerated axons were common only in some of the samples. Most Schwann cells exhibited large nuclei. The configuration of the blood vessels varied both within a sample and between samples, regarding lumen size, BLAT and the appearance of the endothelium. In some vessels reduplication of basal lamina was evident (Fig 3B).

#### Patients with Inflammatory Neuropathy

Extensive axonal degeneration, remyelination of some medium-sized axons, electron-loose Schwann cell cytoplasm, and presence of large vacuoles that sometimes include glycogen bodies in the Schwann cells were the pathological hallmark in this group. Band of Büngner and collagen pockets were observed in all samples. ARS were observed in the cytoplasm of Schwann cells. Macrophages included degradation products, and lucid vacuoles were often observed in Schwann cells and endoneurium. Large Schwann cell nuclei were observed in two samples. The appearance of the blood vessels diverged. In this group several vessels had numerous endothelial cells and periendothelial cell nuclei. A few vessels had a relatively large amount of periendothelial cell processes in the basal lamina area, others had just plain thickening of the basal lamina area including reduplication (Fig 3C).

#### Normal Controls

The endoneurium was occupied by large, medium-sized and small myelinated axons, and unmyelinated fibers which mostly had a 1:1 relation with a Schwann cell. In addition, the nerve samples from individuals in this group exhibited a few degenerated myelinated axons (0–3), bands of Büngner, and collagen pockets in the endoneurium. Lysosomes, autophagosomes, and structures resembling phagophores or autolysosomes were mostly observed in the axons rather than Schwann cells. There was a variation in size of lumen, endothelial area and also basal lamina area in the blood vessels in the samples from normal controls (Fig 3A).

### Electron Microscopy Analysis

#### Unmyelinated Fibers

There was no significant difference in the density of unmyelinated fibers between the three groups, (Table 2). Furthermore, a closer analysis of the unmyelinated fiber density in the five CIAP patients with (median density 39,648 no/mm^2^, range 10,304–56,308) or without neuropathic pain (median density 24,144 no/mm^2^, range 8,210–28,577), revealed no significant difference. The diameter of unmyelinated fibers was significant smaller (*p*<0.0001) in the group with inflammatory neuropathy compared to the other two groups.

#### Endoneurial Microvessels

**Fig 4:**
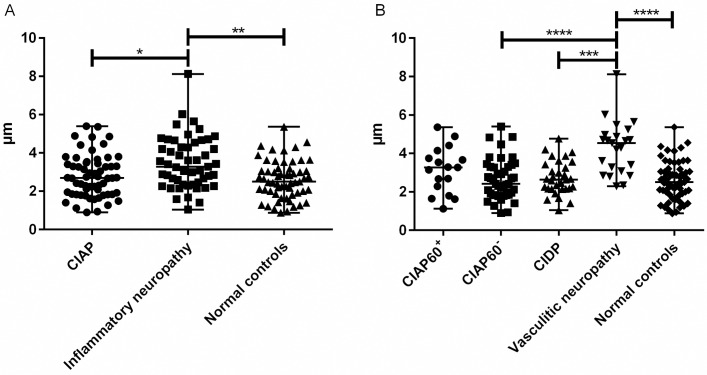
Basal Lamina Area Thickness in Endoneurial Microvessels. Basal lamina area thickness (BLAT) in sural nerves obtained from patients with (a) CIAP, inflammatory neuropathy and normal controls, and (b) in patient subgroups. The BLAT is significantly elevated in patients with inflammatory neuropathy compared to patients with CIAP and normal controls. This elevation is restricted to the subgroup of patients with vasculitic neuropathy and not CIDP. Note that the increase in BLAT in vasculitic neuropathy is not significantly different from the level seen in older patients with CIAP (i.e. those 60 years or older). Vertical bars signify median with range. *p<0.05, **p<0.01, ***p<0.001, ****p<0.0001. CIAP^60+^ = patients with chronic idiopathic axonal polyneuropathy aged 60 years and older. CIAP^60-^ = patients with chronic idiopathic axonal polyneuropathy younger than 60 years (25–59 years old). CIDP = chronic inflammatory demyelinating polyneuropathy.

The BLAT and the endothelial cell area were significantly higher in the group with inflammatory neuropathy than in both the patients with CIAP and normal controls (Table 2 and Fig 4A). There was no significant difference regarding lumen area and the number of endothelial cell nuclei. The number of endothelial cell profile (*p*<0.05) and periendothelial cell nuclei (*p*<0.001) was significantly higher in the patients with inflammatory neuropathy compared to normal controls.

#### Subgroup Analysis of Endoneurial Microvessels

Subgroup analysis of patients with CIAP 60 years or older (CIAP^60+^) and patients with CIAP younger than 60 years (CIAP^60-^) showed no significant difference in BLAT, lumen area or endothelial cell area compared to normal controls, though we found a trend towards a larger median BLAT (3.3 μm) in patients with CIAP^60+^ than normal controls (median BLAT 2.5 μm) (p = 0.08). The median BLAT and the median endothelial cell area in the patients with vasculitic neuropathy showed significantly higher values vs. normal controls (*p*<0.0001) (Fig 4B).

#### The Density and Location of Autophagy-Related Structures

**Fig 5:**
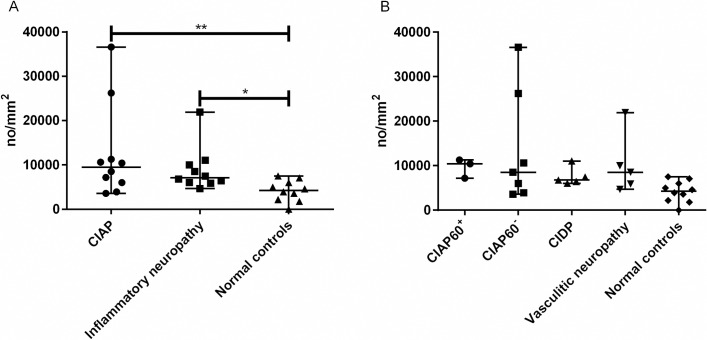
Endoneurial Density of Autophagy-Related Structures in Sural Nerves. Endoneurial density of autophagy-related structures in sural nerves obtained from (a) patients with CIAP, inflammatory neuropathy and normal controls and (b) in patient subgroups. The density of ARS is elevated in CIAP and inflammatory neuropathy compared to the normal controls. Inflammatory neuropathy consists of patients with CIDP and those with vasculitic neuropathy. Vertical bars signify median with range. *p<0.05, ** p<0.01, *** p<0.001, **** p<0.0001. CIAP^60+^ = patients with chronic idiopathic axonal polyneuropathy aged 60 years and older. CIAP^60-^ = patients with chronic idiopathic axonal polyneuropathy younger than 60 years (25–59 years old). CIDP = chronic inflammatory demyelinating polyneuropathy.

The median density of ARS in the sural nerve was significantly higher in patients with CIAP (*p*<0.01) and in patients with inflammatory neuropathy (*p*<0.05) compared to normal controls. The difference was more prominent in patients with CIAP than those with inflammatory polyneuropathy (Table 2 and Fig 5A). Subgroup analysis of patients with CIAP^60+^ and CIAP^60-^ revealed no significant difference with regards to density of ARS compared to controls (Fig 5B).

### Ultrastructural location of autophagy-related structures within sural nerve

**Fig 6:**
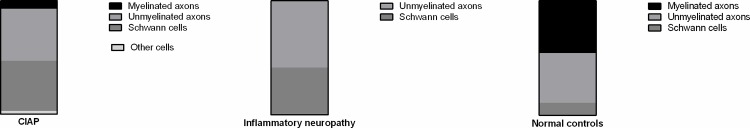
Proportion of Autophagy-Related Structures Found in Respective Specific Location in Sural Nerves of Patients with CIAP, Inflammatory Neuropathy and Healthy Controls.

A significant higher proportion of the total number of ARS was found in the Schwann cells (SC) in peripheral nerves of patients with CIAP vs. normal controls (Table 3, Fig 6); the high proportion occurred specifically in the SC of unmyelinated fibers (*p* = 0.001; Table 3). In myelinated axons, however, the proportion of ARS was lower in samples taken from CIAP and inflammatory neuropathy vs. normal controls (Table 3, Fig 6).

**Table 3:** Specific Location of Autophagy-Related Structures in Sural Nerves of Patients with CIAP, Inflammatory Neuropathy and Normal Controls.

	CIAP *n = 10*	Inflammatory neuropathy *n = 10*^a^	Normal controls *n = 9*
**All Schwann cells (SC)**	0.44** (0.36–0.57)	0.41* (0.22–0.58)	0.10 (0.03–0.24)
**SC of myelinated axons**	0.17 (0.0–0.31)	0.13 (0.05–0.31)	0.67 (0.0–1.0)
**SC of unmyelinated axons**	0.46*** (0.22–0.66)	0.24 (0.0–0.48)	0.0 (0.0–0.0)
**Empty SC**	0.27 (0.09–0.51)	0.40 (0.16–0.65)	0.0 (0.0–0.33)
**Myelinated axons**	0.07* (0.0–0.11)	0.0** (0.0–0.07)	0.42 (0.24–0.71)
**Unmyelinated axons**	0.46 (0.36–0.58)	0.58 (0.27–0.65)	0.40 (0.14–0.58)
**Other cells**^**b**^	0.03 (0.0–0.07)	0.0 (0.0–0.13)	0.0 (0.0–0.0)

Patients and controls with autophagy-related structures are presented in the table.

CIAP = Chronic idiopathic axonal polyneuropathy

Values are presented as median and the 25^th^-75^th^ percentiles of the proportion of autophagy-related structures in each sample.

*p<0.05

**p<0.01 vs. normal controls

^a^Data lacking for one inflammatory control

^b^Other cells comprise of a summation of fibroblasts and endothelial cells

### Influence of age on BLAT and autophagy

**Fig 7:**
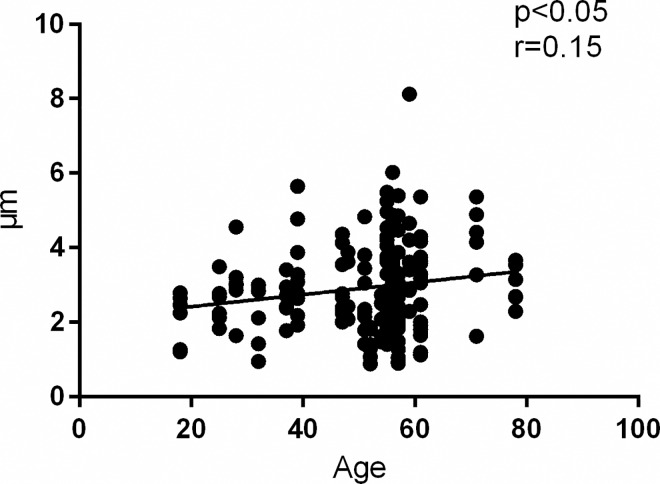
Correlation Analysis of Basal Lamina Area Thickness vs. Age. Correlation analysis of BLAT vs. age in all analyzed nerve samples in the total study population of patients with CIAP, inflammatory neuropathy and normal controls. Basal lamina area thickness correlates positively with age.

There was a significant correlation (*p*<0.05, r = 0.15) between BLAT and age in the entire study population (Fig 7). The density of ARS in sural nerves did not correlate with age.

## Discussion

The main findings of this study were a lack of sural nerve endoneurial microangiopathy markers in the entire cohort of patients with CIAP, and the identification of significant levels of ARS in sural nerves of patients with CIAP and inflammatory neuropathy vs. normal controls.

In the present study, we could not corroborate results of Teunissen and colleagues [39] detecting signs of microangiopathy in vasa nervorum of sural nerves as a cause of CIAP, since we did not find any significant difference in endoneurial BLAT and a number of other markers of microangiopathy in sural nerves of patients with CIAP compared to the normal controls. This earlier ultra-structural study of endoneurial vessels in sural nerves [39] reported a larger BLAT, endothelial cell area and higher number of endothelial cell nuclei in patients with CIAP compared to patients with hereditary motor sensory neuropathy (HMSN) type II and autopsy controls, whereas the BLAT in patients with CIAP and those with diabetic neuropathy were found to be in the same range [39]. A possible explanation for our divergent results compared with the corroborative evidence for the presence of microangiopathy in sural nerves reported by Teunissen and colleagues [39], could be that our CIAP patients (aged 25–78; mean 54.9 years), were younger than those examined by Teunissen (mean age 63 years). Furthermore, we did find a correlation between BLAT and age in the whole cohort of patients and controls, which is also in accordance with results of the Jacobs and Love study [43]. The significance of this age-related increase in BLAT i.e. of age-dependent microangiopathy in endoneurial blood vessels for the pathogenesis of CIAP is not clear, since we did not find a statistically significant increase in BLAT in CIAP patients older than 60 years.

The patient group that unexpectedly did show signs of endoneurial microangiopathy as reflected by increased BLAT and endothelial cell hypertrophy/hyperplasia turned out to be those with vasculitic neuropathy. In subgroup analysis, the patients with vasculitic neuropathy had significantly increased BLAT and endothelial cell area compared to normal controls. To ascertain that the increased BLAT in vasculitic neuropathy is not merely due to a cellular infiltration, we excluded those vessels with prominent periendothelial cytoplasmic structures with little actual basal lamina as these microstructural changes may indirectly signify the occurrence of inflammatory cells in the basal lamina area zone. Despite this selection, the values for BLAT of patients with vasculitic neuropathy remained significantly elevated. Another possible explanation for large BLATs in vasculitic neuropathy could be edema, but we did not observe any apparent structural gaps in the endothelial junctions to support this hypothesis, and one patient actually had a reduplication of the basal lamina.

Finally, the lack of previous published reports on possible increased BLAT in vasculitic neuropathy may be due to the fact that changes in vessel structure in patients with vasculitic neuropathy have mainly been studied in epineurial [44, 45] rather than endoneurial vessels which were the blood vessels studied by us. When reviewing the literature we could only identify a single case report of a patient with Wegener’s granulomatosis where the endoneurial vessels of the sural nerve was studied and shown to have thickened and reduplicated basal lamina compared to a control subject [46]. Similar description of structural abnormalities in the endoneurial vessels is found in a case report of a patient with panarteritis nodosa [47].

The other significant finding of this study was a significantly higher density of ARS in sural nerves of patients with CIAP and inflammatory neuropathy compared to normal controls. As far as we know, this is the second report of existence of ARS in human peripheral nerve [33], and the first report of ARS being located in sural nerves of patients with CIAP and inflammatory neuropathy.

The role of the autophagy pathway and the potential activation of the autophagy machinery in pathological processes are not fully explored in the human peripheral nerve. In animal models with metabolic abnormalities, increased number of ARS [48], as well as an up-regulated autophagy pathway [49] has been reported. It has been suggested that autophagy has a protective role in preventing neuropathy in a rat model with metabolic risk factors [49]. Autophagy structures are up-regulated by myelinating Schwann cells and participate in the degradation of myelin proteins and lipids after nerve injury [50]. Human neuroblastoma cells that were exposed to sera from patients with diabetes and neuropathy showed an increased rate of autophagy, which was not the case for the cells exposed to sera from diabetes patients without neuropathy [51].

In agreement with the results of Osman and colleagues [33], our results indicate that the autophagy machinery is present in human peripheral nerve. Furthermore, we show that although ARS are encountered in sural nerves both of patients with CIAP and those with inflammatory neuropathy, the ARS are more abundantly encountered in CIAP than in inflammatory neuropathy. In patients with CIAP and inflammatory neuropathy the ARS were significantly more abundant in Schwann cells compared to normal controls. However in patients with CIAP, but not those with inflammatory neuropathy, the ARS were preferentially located within Schwann cells of unmyelinated axons. On the other hand, and similar to patients with inflammatory neuropathy, the ARS in patients with CIAP were less likely to be found within myelinated axons, in comparison with normal controls. The cause of the differences with regards to level and location of ARS in sural nerves of patients with CIAP vs. normal controls is not known. If the autophagy process indeed constitutes the cells´ attempt at counteracting axonal degeneration [50], then the relative scarcity of ARS in myelinated axons of patients with CIAP may be indicative of myelinated axons´ inability to withstand axonal damage. Naturally, we cannot exclude the possibility that the lower number of ARS in the myelinated axons in CIAP patients, merely is due to axonal degeneration.

Interestingly, we did not find a correlation between the density of ARS and age, so the activated autophagy pathway does not seem to be a normal age-related process. Nor did we find a correlation between the duration of the disease and the density of ARS, suggesting that the activation of the autophagy pathway appears to be an early ongoing process in CIAP and not merely an end-stage one. Naturally, we cannot provide evidence towards a causal relationship neither between autophagy nor the lack thereof with axonal degeneration specific to CIAP, since we saw a similar pattern (though to a milder extent) of autophagy alterations in patients with inflammatory neuropathy.

Pain is a common symptom in patients with CIAP [52]. Patients with CIAP may have small fiber involvement [53]. In the present study, we could not show any loss of unmyelinated fibers in patients with CIAP compared to patients with inflammatory neuropathy and healthy controls. Furthermore, no significant difference was detected when comparing unmyelinated fiber density in the CIAP patients with (n = 5) pain or without (n = 5) pain. All the patients with pain had performed a quantitative sensory testing of their temperature thresholds and in all cases the results were abnormal. This could indicate that the small fibers are functionally rather than structurally affected in patients with CIAP with neuropathic pain.

Limitations with our study are the few patients and controls included as well as the retrospective study set up. However, sural nerve biopsy is an invasive procedure with risk of residual damage which limits the number of patients that are put through this procedure nowadays as part of the work up of unclear neuropathies. Hence, we considered it of great interest to perform this study with the existing limited material. A limitation with the retrospective study set up is selection bias, since the final clinical diagnosis of CIAP, CIDP and vasculitic neuropathy was determined by the neuropathological evaluation of the sural biopsy. Another limitation is the lack of information about smoking habits and possible metabolic or cardiovascular risk factors and associated comorbidities for the normal controls.

Patients with CIAP are a heterogeneous group, in part due to the difficulty in differentiating sporadic HMSN type 2 from CIAP. Our CIAP patients, much like patients with HMSN, were relatively young, especially in the case of a single patient with CIAP being 25 years old. However, we do not suspect this patient to have a HMSN type 2 since the clinical phenotype does not fit the disease.

## Conclusion

To conclude, we did not find evidence of microangiopathy in endoneurial blood vessels in sural nerves in patients with CIAP. The density of autophagy-related structures in the sural nerve was significantly increased in an age-independent manner in patients with CIAP, adding CIAP to the list of neuromuscular disorders which have been shown to be associated with an alteration in the autophagic process in target tissue. It is as yet not clear if the altered autophagy pathway is a consequence or a cause of the neuropathy in CIAP. Further immunohistochemistry and protein analysis studies are required to better understand the process of autophagy and its role in seemingly idiopathic chronic axonal neuropathy.

## Supporting Information

S1 File This is the dataset of all the data used for this manuscript. (XLSX)
